# Lymphocyte Polarization During Immune Synapse Assembly: Centrosomal Actin Joins the Game

**DOI:** 10.3389/fimmu.2022.830835

**Published:** 2022-02-11

**Authors:** Chiara Cassioli, Cosima T. Baldari

**Affiliations:** Department of Life Sciences, University of Siena, Siena, Italy

**Keywords:** immune synapse, centrosome polarization, F-actin clearance, T lymphocytes, B lymphocytes

## Abstract

Interactions among immune cells are essential for the development of adaptive immune responses. The immunological synapse (IS) provides a specialized platform for integration of signals and intercellular communication between T lymphocytes and antigen presenting cells (APCs). In the T cell the reorganization of surface molecules at the synaptic interface is initiated by T cell receptor binding to a cognate peptide-major histocompatibility complex on the APC surface and is accompanied by a polarized remodelling of the cytoskeleton and centrosome reorientation to a subsynaptic position. Although there is a general agreement on polarizing signals and mechanisms driving centrosome reorientation during IS assembly, the primary events that prepare for centrosome repositioning remain largely unexplored. It has been recently shown that in resting lymphocytes a local polymerization of filamentous actin (F-actin) at the centrosome contributes to anchoring this organelle to the nucleus. During early stages of IS formation centrosomal F-actin undergoes depletion, allowing for centrosome detachment from the nucleus and its polarization towards the synaptic membrane. We recently demonstrated that in CD4^+^ T cells the reduction in centrosomal F-actin relies on the activity of a centrosome-associated proteasome and implicated the ciliopathy-related Bardet-Biedl syndrome 1 protein in the dynein-dependent recruitment of the proteasome 19S regulatory subunit to the centrosome. In this short review we will feature our recent findings that collectively provide a new function for BBS proteins and the proteasome in actin dynamics, centrosome polarization and T cell activation.

## Introduction

The immunological synapse (IS) is a specialized interface formed by T lymphocytes with antigen presenting cells (APCs) bearing a cognate peptide-major histocompatibility complex (pMHC) that allows for information exchange and execution of effector functions (1, 2). In its canonical configuration the IS is characterized by concentric Supra Molecular Activation Clusters (SMACs) differing in composition and function (3, 4). Ligand-bound antigen T cell receptors (TCRs) and associated signaling molecules occupy the central SMAC (cSMAC), which is surrounded by a peripheral SMAC (pSMAC) enriched in adhesion molecules, such as the integrin LFA-1, and an outer distal SMAC (dSMAC), where filamentous actin (F-actin) and CD45 are concentrated (4, 5). Immune cell interactions rely on continuous cytoskeleton remodeling events, which not only shape the T cell-APC interface, but also asymmetrically distribute molecules and organelles within the lymphocyte, leading to the establishment of transient polarity (6, 7). Cytoskeleton remodeling is one of the earliest events induced by TCR signaling (8) and culminates in the formation of a synaptic F-actin ring, allowing the centrosome to polarize to the IS (9) together with the Golgi apparatus, endosomal and secretory compartments, multivesicular bodies (MBVs) and mitochondria (10–14). This polarized configuration is instrumental in the directional delivery of TCR^+^ recycling endosomes to the synaptic membrane (15–17) that thus is refilled of signaling-competent receptors as exhausted TCRs are internalized to be sorted for recycling or lysosomal degradation (18, 19). Alternatively, post-endocytic TCRs are directed to MVBs and incorporated in intraluminal vesicles (ILVs), which are released into the synaptic cleft as exosomes and taken up by APCs (20). Mitochondria are also mobilized towards the APC contact site, where they contribute to IS formation and TCR signaling by providing a local source of ATP and modulating the concentration of intracellular calcium (13, 21, 22).

The rapid repositioning of the centrosome following F-actin depletion from the IS center highlights a spatiotemporal coordination between the actin and microtubule cytoskeletons during IS formation. Interestingly, recent studies have unveiled a new feature of the interplay between F-actin dynamics and centrosome repositioning during IS assembly, demonstrating that the balance between F-actin polymerization and depolymerization at the centrosome is crucial for its ability to untether from the nucleus and polarize to the IS. Here, we will summarize how actin dynamics at the synaptic and centrosomal areas regulate IS assembly. We will then describe the emerging role of the ubiquitin-proteasome system (UPS) in centrosomal F-actin remodeling, focusing on our recent findings that identify the ciliary protein Bardet-Biedl Syndrome 1 (BBS1) as a new regulator of T cell polarity during IS formation (23).

## Regulation of Centrosome Polarization by the Actin Cytoskeleton

### Synaptic F-Actin Controls Centrosome Repositioning to the IS and Microtubule-Driven Exocytosis

TCR engagement by cognate pMHC promotes profound cytoskeletal changes, achieved *via* the coordinated reorganization of the actin and microtubule cytoskeletons at the IS. Actin remodeling is triggered within seconds after TCR stimulation (8) and precedes centrosome translocation towards the IS (9). Imaging cortical actin at the T cell IS using super-resolution microscopy techniques has revealed the coexistence of distinct actin-based networks in the three concentric regions, or SMACs, featured by the IS: from the outer edge to the center, a lamellipodial branched actin network (dSMAC), the lamellar acto-myosin network (pSMAC), a network consisting of actin foci spread throughout the dSMAC and pSMAC, and an hypodense actin network at the center (cSMAC) [synaptic actin networks are extensively reviewed in (24, 25)]. Another category of actin-based structures, the microvilli, has been described at the T cell surface (26). Although these microvillar extensions are a feature of resting T cells, their study has been recently extended to activated cells based on the observation that TCRs cluster at microvillar tips (27). T cell microvilli had been initially considered as sensors during antigen survey on APCs. However, the discovery of T cell microvillar particles (TMP) deposited on the APC surface has suggested the possibility that they may act as “immunological synaptosomes” that deliver a new class of membrane vesicles as a means of intercellular communication (28, 29).

TCR signaling is the main extrinsic cue for centrosome reorientation to the IS (30), with the second messenger diacylglycerol (DAG), which forms a gradient centered at the cSMAC, acting as a polarity determinant (31, 32). Multiple signaling pathways initiated at the IS by the TCR, the integrin LFA-1 and the co-stimulatory receptor CD28 coordinate the activation of several actin-regulatory proteins that promote F-actin polymerization, feeding back for optimal TCR signaling, integrin activation and T cell spreading over the APC (33, 34). Interestingly, both disruption of synaptic actin networks and depletion of F-actin nucleators (e.g. the formins diaphanous 1 and formin-like-1) result not only in impaired centrosome mobilization but also in defective TCR signaling, suggesting an indirect role of actin in centrosome repositioning. The physical interaction between microtubule (+)ends and branched F-actin network at the IS periphery, which is mediated by molecular linkers that include the IQ domain-containing GTPase-activating protein, ezrin and Cdc42-interacting protein 4 (35–37), provides an additional function for actin in centrosome polarization by generating tension on microtubules.

Centrosome repositioning is considered a hallmark of T cell polarity during IS assembly. The reorientation of this organelle is accompanied by the polarization of other intracellular compartments, including secretory and recycling endosomes that require microtubule tracks for delivery to the IS and focalized exocytosis. Emerging evidence indicates an early participation of F-actin in polarized recycling. F-actin polymerizes at recycling endosomes through the assistance of the F-actin regulator Wiskott-Aldrich Syndrome protein and SCAR Homology (WASH) and its partner FAM21 to help membrane scission of nascent vesicles carrying recycling cargo, including the TCR, LFA-1, CD28 and the glucose transporter GLUT-1. These receptors exploit the recycling pathway to accumulate at the IS, where they participate in mature IS formation and maintenance, as well as in the metabolic reprogramming of activated T cells (38). The role of actin in the final steps of vesicle fusion and exocytosis at the IS is less clear-cut. Initially, cortical actin has been regarded as a barrier that prevents vesicle exocytosis. Consistent with this view, in T_H_ cells the focalized release of IFN-γ at the IS was found to be impaired in Cdc42-silenced T cells due to their failure to form a synaptic actin ring (39). Moreover, lattice-light-sheet microscopy of CTL-target cell conjugates showed that at mature synapses F-actin is cleared from the cSMAC before lytic granule secretion, and that lytic granule release triggers F-actin recovery at the lytic synapse blocking further lytic granule exocytosis and serial killing (40, 41). However, a more in-depth analysis of actin dynamics at the lytic synapse using super-resolution microscopy has revealed that the cSMAC is not entirely free from F-actin, but rather occupied by a hypodense actin network (42–44). Upon cell activation, holes of a size compatible with the access of lytic granules to the plasma membrane for docking and fusion have been observed (42, 44). Hence actin plays a dual role during secretion depending on the maturation stage of the lytic synapse: at immature synapses a dense actin cortex blocks secretion, while in mature, actin-hypodense synapses nanoscale actin filament dynamics fine-tunes regulated lytic granule exocytosis (45).

### A Centrosome-Associated F-Actin Pool Contributes to Lymphocyte Polarization

**Figure 1:**
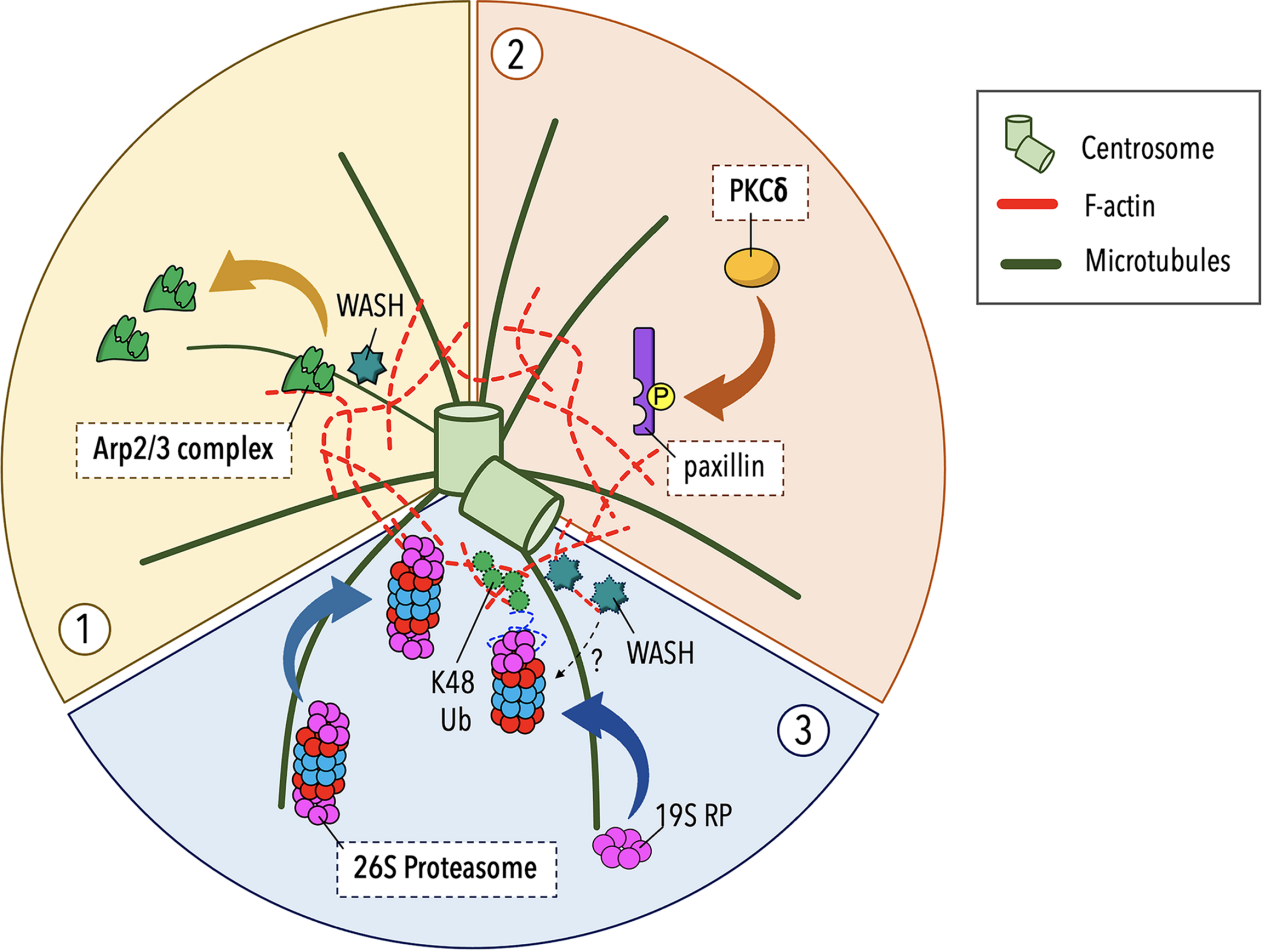
Regulation of centrosomal F-actin clearance in lymphocytes. Recent studies carried out on B and T lymphocytes have implicated three discrete pathways in centrosomal F-actin clearance and centrosome polarization during early stages of IS assembly. (**1**) In B lymphocytes the recruitment of the branched F-actin nucleator Arp2/3 to the IS upon BCR activation leads to a local depletion of centrosome-associated Arp2/3 that results in reduced F-actin polymerization at the centrosome, allowing for centrosome untethering from the nucleus and its repositioning to the B cell IS (51). (**2**) In T lymphocytes protein kinase C-δ (PKCδ) has been identified as a novel regulator of centrosomal F-actin remodeling, beyond its role in cortical actin reorganization at the IS. Following TCR triggering, PKCδ phosphorylates the scaffold protein paxillin, which localizes at the centrosome where it contributes to centrosome translocation to the T cell IS by promoting a local F-actin clearance through an unknown mechanism (57). (**3**) An alternative pathway, based on the proteolytic activity of a centrosome-associated proteasome, controls F-actin clearance from the centrosome to enable its dissociation from the nucleus and polarization to the nascent IS. This pathway is exploited by both B and T lymphocytes with some cell type-dependent features. In B lymphocytes the intracellular distribution of the proteasome is regulated by the proteasome adapter and scaffold protein Ecm29 during B cell IS formation, allowing a sequential recruitment of the proteasome to the centrosome and then to the IS, which is crucial for F-actin reorganization at both locations (58, 59). In T lymphocytes proteasome-mediated degradation of unknown targets at early stages of IS assembly is dependent on the transport of the 19S regulatory particle (RP) to the centrosome, which is paralleled by an active degradation of K48-linked polyubiquitylated proteins (K48 Ub) (23). The contribution extent as well as the sequential implication of these pathways to the process remain to be elucidated.

Although the centrosome is the major microtubule-organizing center, F-actin and microtubules coexist at the centrosomal area. Proteomic analyses have documented the presence of actin and actin-associated proteins at the centrosome (46–51) and different actin structures have been reported in association with the centrosome and the nucleus in different cell types (52–55). Recent studies carried out on B lymphocytes have revealed that the centrosome is surrounded by a local, cloud-like meshwork of F-actin. A central function of this F-actin pool is to anchor the centrosome to the nucleus *via* the Linker of Nucleoskeleton and Cytoskeleton (LINC) complex (51). Moreover, centrosome-associated F-actin can prevent microtubule growth from the centrosome, as supported by *in vitro* reconstitution assay on purified centrosomes (56), suggesting that synaptic F-actin clearance is not sufficient for centrosome polarization to the IS. Of note, the centrosome-associated F-actin network undergoes a dynamic turnover though repeated cycles of polymerization and depolymerization. Upstream centrosome polarization to the IS, the balance between F-actin assembly and disassembly is tilted towards the latter leading to a local depletion of centrosomal F-actin, which facilitates centrosome detachment from the nucleus and its subsequent translocation towards the IS. In B cells this occurs through a reduced recruitment of the actin nucleator Arp2/3 at the centrosome (**Figure 1**) in favor of its synaptic localization (51).

A more recent study on Jurkat T cell-APC conjugates has demonstrated that centrosome-associated F-actin remodeling is a mechanism controlling centrosome polarization also in T lymphocytes (57). A pathway involving protein kinase C-δ (PKCδ), which is activated downstream TCR engagement, has been proposed to regulate centrosomal F-actin in T cells (**Figure 1**). The PKCδ-mediated phosphorylation of the cytoskeleton adaptor protein paxillin, which is associated with the centrosome under resting and stimulating conditions, has been related to F-actin clearance around the centrosome and its polarization to the IS (57), however the underlying mechanism remains to be elucidated. Since paxillin interacts with several signaling and cytoskeletal proteins (60, 61), it is likely that paxillin directly or indirectly binds to one or more actin regulators. Furthermore, paxillin associates with the microtubule cytoskeleton (62, 63), suggesting that it might play a role in the actin-microtubule interplay that drive centrosome repositioning to the IS. An interesting aspect in the model proposed by Bello-Gamboa et al. is that the reorganization of the actin cytoskeleton at the centrosome occurs in a coordinated fashion with synaptic F-actin remodeling. In fact, PKCδ was found to phosphorylate another substrate, the formin-like molecule FMNL1β, that is recruited to the IS (57), where membrane-bound formins generate bundles of linear actin filaments across the dSMAC (64, 65).

## The Ubiquitin-Proteasome System (UPS) and BBS1 Cooperate in Centrosomal F-Actin Clearance in T Cells

In recent years new, unexpected players have been identified in the mechanisms that regulate centrosomal F-actin clearance and centrosome polarization during IS assembly. We and Ibañez-Vega et al. have implicated the ubiquitin-proteasome system (UPS) in this process (23, 58, 59) (**Figure 1**). The UPS is a major degradation pathway in eukaryotic cells and is responsible for proteolysis of cytosolic proteins regulating a variety of cellular processes. This system consists of ubiquitin ligases that target proteins for degradation by covalently adding ubiquitin to proteasome substrates, allowing for their recognition by the 26S proteasome. The 26S proteasome is a multisubunit complex, composed by the 19S regulatory particle (RP) and the 20S core particle (CP), which identifies, unfolds, and degrades ubiquitinated proteins in an ATP-dependent manner (66). Accumulating evidence suggests a role for the proteasome in centrosome proteostasis (67), and thus in centrosome-related functions, including cell polarity in neurons (68, 69), growth and signaling function of the primary cilium in ciliated cells (70–74), and differentiation and metabolic profile of CD8^+^ T cells (75, 76).

Although the proteasome is largely cytosolic, a proteasomal pool is associated with the centrosome, as witnessed by a local accumulation of proteasome subunits and proteasomal substrates around the centrosome (67). Recently, it has been reported that in B cells proteasome relocalization from the centrosome to the IS is required for the spatiotemporal coordination of centrosomal and synaptic F-actin depolymerization (58, 59). In T lymphocytes a proteomic analysis of centrosomes purified from activated cells has revealed a local enrichment of proteasomal components (77). Consistent with this evidence, we observed that the centrosomal 19S RP pool increases early during IS formation, to progressively return to baseline with IS maturation (23). Additionally, we found that the centrosome failed to polarize to the IS in T cells pre-treated with proteasome inhibitors and that the mislocation of the centrosome in these cells is paralleled by F-actin accumulation at the centrosome, supporting a role for the centrosomal proteasome in IS formation. The mechanisms linking the centrosomal proteasome to the local F-actin pool remain unknown. While actin depletion is regulated by the balance in the depolymerization and *de novo* synthesis of actin filaments, proteasome-regulated and centrosome-associated actin nucleators, such as Arp2/3 and its activator WASH [assembly and activity of the WASH complex are extensively reviewed in (78)], which become depleted during IS assembly (23), may represent potential targets of the centrosomal proteasome. Particularly interesting candidates are the proteasome-regulated E3 ligase TRIM27, which activates WASH through K63 mono-ubiquitination (79), and the centriolar satellite protein PCM1 (80), which recruits Arp2/3 and WASH to the T cell centrosome (50). Whether these are actual targets of the centrosomal proteasome, and the relative contribution of degradation versus changes in subcellular localization to the centrosomal depletion of Arp2/3 and WASH as well as to the resulting local decrease in F-actin during IS formation, are important issues to be addressed.

An expected twist in the proteasome-dependent regulation of centrosomal F-actin was the identification of the ciliopathy-related protein BBS1 as a novel player in T cell IS assembly (23). The BBS complex, or BBSome, is an evolutionary conserved octameric complex that regulates cilia-based signaling pathways by acting as an adaptor between the Intraflagellar transport-B (IFT-B) complex and membrane proteins, of which activated G protein-coupled receptors (GPCRs) are the main group, for their ciliary exit (81, 82). Several ciliogenesis proteins and ciliary signaling pathways are important regulators of different steps in T cell IS assembly (83, 84) and the BBSome core component BBS1 is no exception. In our recent work we demonstrated that BBS1 controls a key event in IS formation, i.e. centrosome polarization, on which the synaptic recruitment of endosomal TCRs and accumulation of tyrosine phosphoproteins depend. Having ruled out an early TCR signaling defect as the cause of the inability of the centrosome to polarize to the IS in BBS1-depleted T cells, we hypothesized alternative explanations. Centrosomal F-actin clearance appeared an interesting possibility, since in BBS4-, BBS6- or BBS9-depleted cells ciliogenesis is compromised due to increased F-actin polymerization (85). Indeed, we found that F-actin failed to be cleared from the centrosome in the absence of BBS1 and that the persistence of a F-actin meshwork at the centrosome was paralleled by a local accumulation of WASH (23). Our results identify a novel cilium-independent function for BBS1 in the clearance of centrosomal F-actin, due at least in part to local depletion of WASH. This is expected to result in impaired F-actin nucleation that is not able to counterbalance the continuous depolymerization of pre-existing filaments, eventually leading to centrosome disengagement from the nucleus and its polarization to the IS. A more in-depth investigation of the molecular mechanism by which BBS1 controls centrosomal F-actin dynamics in T cells revealed that, consistent with the BBSome function in dynein-dependent retrograde transport of ciliary cargo to the base of the cilium (82), BBS1 acts as a dynein adaptor coupling the 19S RP with dynein allowing for its transport to the centrosome. Consistent with this function, a lesser recruitment of 19S RP to the centrosome and an increased centrosomal accumulation of K48-polyubiquitylated proteins were observed in conjugates of BBS1-deficient T cells (23).

**Figure 2:**
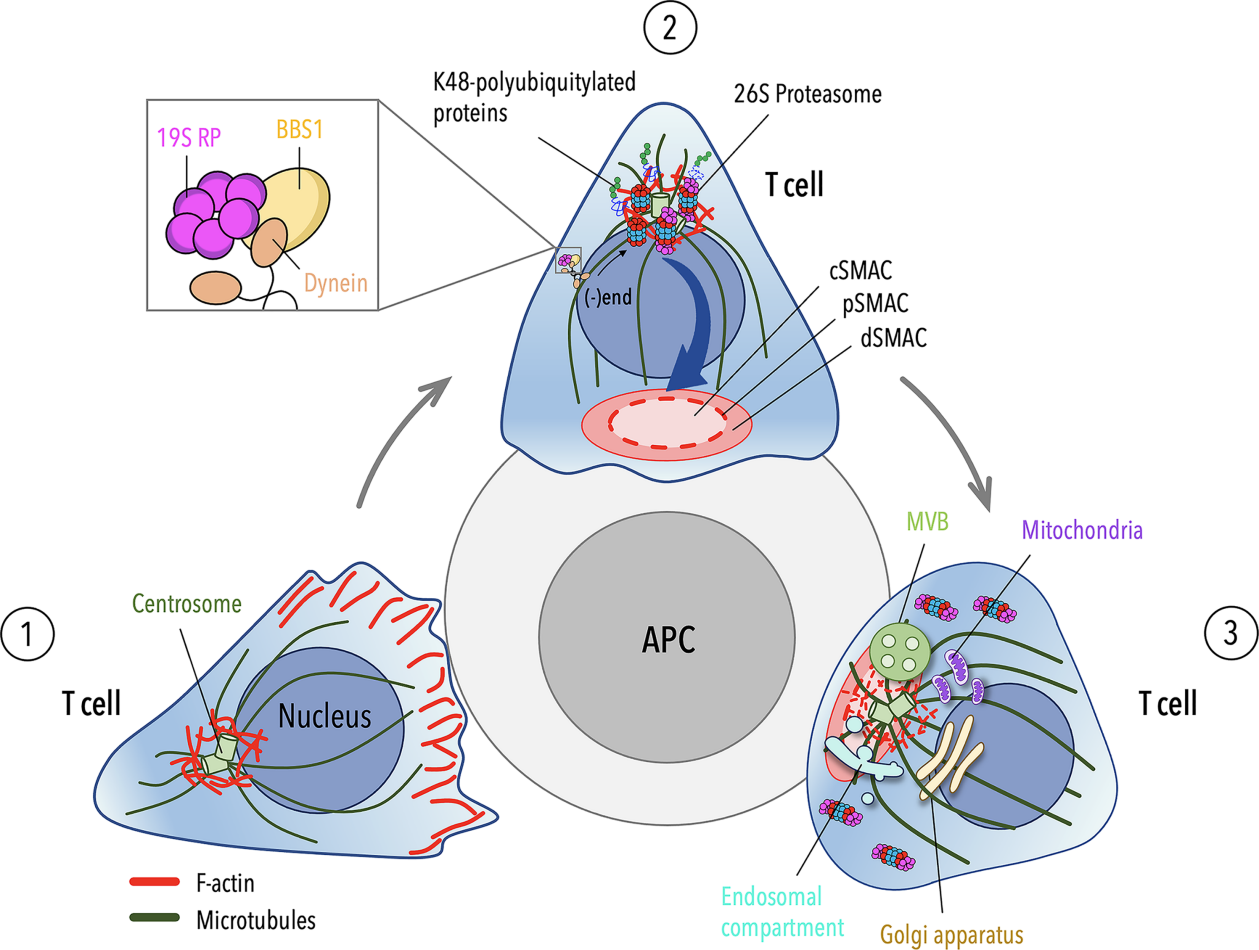
Cytoskeleton-driven events leading to centrosome polarization during T cell IS assembly. (**1**) Migrating T cells exhibit an actin-rich leading-edge and a uropod protruding from the rear of the cell. The centrosome localizes at the trailing edge of the cell and is tethered to the nucleus by a F-actin pool associated with the centrosome. (**2**) Upon antigen presenting cell (APC) encounter, actin polymerizes and accumulates at the contact site to drive the spreading of the T cell across the APC. On the contrary, centrosomal F-actin undergoes depletion during IS formation to allow for centrosome detachment from the nucleus and its translocation to the nascent IS. Centrosome-associated proteasome, which is endowed of enhanced proteolytic activity within the first minute of IS assembly (23), plays a key role in this process by shifting the balance between polymerization and depolymerization of centrosomal F-actin towards the latter, thus leading to its clearance from the centrosome. The activity of the centrosome-associated proteasome is controlled by the ciliary protein BBS1, which regulates the traffic of the proteasome 19S regulatory particle (RP) to the centrosome by coupling 19S RP with the molecular motor dynein. (**3**) The centrosome moves from a site proximal to the IS close to the plasma membrane as cortical F-actin reorganizes into the distal SMAC (dSMAC), leaving a hypodense central SMAC (cSMAC) actin network at the mature IS. Microtubule tethering to the actin network (35–37, 86), capture of (+)ends by dynein at the synaptic membrane and microtubule dynamics (77, 87–90) contribute to the full polarization of the centrosome. In association with the centrosome, other organelles, such as the Golgi apparatus, the endosomal and recycling compartments, multivesicular bodies (MVBs) and mitochondria, converge to the IS to sustain TCR signaling and metabolic reprogramming leading to T cell activation.

Taken together, our findings indicate that, similar to B cells, T cells exploit a proteasome-mediated pathway for centrosomal F-actin clearance, allowing for centrosome polarization to the IS. In this context, the ciliary protein BBS1 regulates the local activity of a centrosome-associated proteasome by coupling the 19S RP to dynein to allow for its retrograde transport to the centrosome (**Figure 2**).

## Conclusions and Future Directions

While cortical F-actin remodeling has been long considered sufficient for centrosome polarization (40), the existence of a F-actin meshwork around the centrosome, the clearance of which is required for centrosome polarization during IS assembly, has added a new level of complexity to the regulation of this process. Currently, three major pathways have been implicated in the depletion of the centrosome-associated F-actin pool and centrosome polarization in lymphocytes. Despite these recent advances, major questions remain to address. For example, whether at some point these pathways intersect converging on shared regulators, or whether they are distinct pathways that sequentially participate in centrosome repositioning to the IS. Imaging studies of the kinetics of centrosome polarization suggested that this process consists of two steps: a relative fast reorientation of the centrosome towards the IS, followed by a slower approach of the centrosome to the synaptic membrane (87). One possibility might be that the dynein- and -BBS1-mediated centrosomal recruitment of either the proteasome or its regulatory component occurs early during centrosome polarization promoting a partial centrosomal F-actin depletion, concomitant with centrosome mobilization to the IS. When the centrosome is in the correct orientation relative to the IS, the synaptic recruitment of the Arp2/3 complex or the PKCδ-dependent phosphorylation of paxillin might complete centrosomal F-actin clearance allowing a full polarization of the centrosome towards the synaptic membrane. Since centrosome polarization can be triggered both by TCR and non-TCR signals (91, 92), we can speculate that a rapid translocation of the centrosome to the IS is driven by TCR-independent signals or minimal TCR signals, while a slower movement of the centrosome towards the maturing IS is tightly controlled by the TCR signaling pathway.

Another major challenge due to the tight squeezing of organelles around the centrosome in the cytoplasm-poor lymphocyte is to image centrosomal F-actin in T cell-APC conjugates at a super-resolution level to confirm that the changes observed in the centrosomal F-actin meshwork are restricted to the centrosome without any involvement of membrane-bound organelles, such as the pericentrosomal endosomal compartment, which is also a site of active actin dynamics. Additionally, super-resolution live-cell imaging is expected to elucidate the interplay of centrosomal F-actin and microtubules during their coordinated reorganization. These considerations suggest novel, exciting directions to be explored in order to better characterize the role of F-actin in centrosome polarization and to identify new regulators of cell polarity during IS formation. Our implication of a BBSome component in IS assembly in the non-ciliated T cell, which further supports the homology of this structure with the primary cilium (83, 84) opens the possibility that other BBS proteins or ciliogenesis proteins functionally related to the BBSome may participate in these processes.

## Author Contributions

CC and CB wrote the manuscript and conceptualized the figures. CC prepared the figures. All authors substantially, directly, and intellectually contributed to the article and approved the submitted version.

## Funding

The work discussed in this review was carried out with the support of AIRC (IG 2017- ID 20148), Ministero dell’Istruzione, dell’Università e della Ricerca (Grant PRIN bando 2017 – 2017FS5SHL), and ERC Synergy Grant 951329 to CB.

## Conflict of Interest

The authors declare that the research was conducted in the absence of any commercial or financial relationships that could be construed as a potential conflict of interest.

## Publisher’s Note

All claims expressed in this article are solely those of the authors and do not necessarily represent those of their affiliated organizations, or those of the publisher, the editors and the reviewers. Any product that may be evaluated in this article, or claim that may be made by its manufacturer, is not guaranteed or endorsed by the publisher.

## References

[1] Harwood NE Batista FD. Early Events in B Cell Activation. Annu Rev Immunol (2010) 28:185–210. doi: 10.1146/annurev-immunol-030409-101216 20192804

[2] Dustin ML Chakraborty AK Shaw AS. Understanding the Structure and Function of the Immunological Synapse. Cold Spring Harb Perspect Biol (2010) 2:a002311. doi: 10.1101/cshperspect.a002311 20843980 PMC2944359

[3] Monks CR Freiberg BA Kupfer H Sciaky N Kupfer A. Three-Dimensional Segregation of Supramolecular Activation Clusters in T Cells. Nature (1998) 395:82–6. doi: 10.1038/25764 9738502

[4] Freiberg BA Kupfer H Maslanik W Delli J Kappler J Zaller DM. Staging and Resetting T Cell Activation in SMACs. Nat Immunol (2002) 3:911–7. doi: 10.1038/ni836 12244310

[5] Stinchcombe JC Bossi G Booth S Griffiths GM. The Immunological Synapse of CTL Contains a Secretory Domain and Membrane Bridges. Immunity (2001) 15:751–61. doi: 10.1016/s1074-7613(01)00234-5 11728337

[6] Gomez TS Billadeau DD. T Cell Activation and the Cytoskeleton: You Can’t Have One Without the Other. Adv Immunol (2008) 97:1–64. doi: 10.1016/S0065-2776(08)00001-1 18501768

[7] Huse M Le Floc’h A Liu X. From Lipid Second Messengers to Molecular Motors: Microtubule-Organizing Center Reorientation in T Cells. Immunol Rev (2013) 256:95–106. doi: 10.1111/imr.12116 24117815 PMC4595039

[8] Locard-Paulet M Voisinne G Froment C Goncalves Menoita M Ounoughene Y Girard L. LymphoAtlas: A Dynamic and Integrated Phosphoproteomic Resource of TCR Signaling in Primary T Cells Reveals ITSN2 as a Regulator of Effector Functions. Mol Syst Biol (2020) 16:e9524. doi: 10.15252/msb.20209524 32618424 PMC7333348

[9] Stinchcombe JC Majorovits E Bossi G Fuller S Griffiths GM. Centrosome Polarization Delivers Secretory Granules to the Immunological Synapse. Nature (2006) 443:462–5. doi: 10.1038/nature05071 17006514

[10] Geiger B Rosen D Berke G. Spatial Relationships of Microtubule-Organizing Centers and the Contact Area of Cytotoxic T Lymphocytes and Target Cells. J Cell Biol (1982) 95:137–43. doi: 10.1083/jcb.95.1.137 PMC2112358 6982900

[11] Kupfer A Swain SL Singer SJ. The Specific Direct Interaction of Helper T Cells and Antigen-Presenting B Cells. II. Reorientation of the Microtubule Organizing Center and Reorganization of the Membrane-Associated Cytoskeleton Inside the Bound Helper T Cells. J Exp Med (1987) 165:1565–80. doi: 10.1084/jem.165.6.1565 PMC2188362 2953845

[12] Varma R Campi G Yokosuka T Saito T Dustin ML. T Cell Receptor-Proximal Signals Are Sustained in Peripheral Microclusters and Terminated in the Central Supramolecular Activation Cluster. Immunity (2006) 25:117–27. doi: 10.1016/j.immuni.2006.04.010 PMC1626533 16860761

[13] Quintana A Schwindling C Wenning AS Becherer U Rettig J Schwarz EC. T Cell Activation Requires Mitochondrial Translocation to the Immunological Synapse. Proc Natl Acad Sci USA (2007) 104:14418–23. doi: 10.1073/pnas.0703126104 PMC1964825 17726106

[14] Bustos-Morán E Blas-Rus N Martín-Cófreces NB Sánchez-Madrid F. Orchestrating Lymphocyte Polarity in Cognate Immune Cell-Cell Interactions. Int Rev Cell Mol Biol (2016) 327:195–261. doi: 10.1016/bs.ircmb.2016.06.004 27692176 PMC5115617

[15] Soares H Lasserre R Alcover A. Orchestrating Cytoskeleton and Intracellular Vesicle Traffic to Build Functional Immunological Synapses. Immunol Rev (2013) 256:118–32. doi: 10.1111/imr.12110 24117817

[16] Finetti F Cassioli C Baldari CT. Transcellular Communication at the Immunological Synapse: A Vesicular Traffic-Mediated Mutual Exchange. F1000Research (2017) 6:1880. doi: 10.12688/f1000research.11944.1 29123650 PMC5657015

[17] Onnis A Baldari CT. Orchestration of Immunological Synapse Assembly by Vesicular Trafficking. Front Cell Dev Biol (2019) 7:110. doi: 10.3389/fcell.2019.00110 31334230 PMC6616304

[18] Vardhana S Choudhuri K Varma R Dustin ML. Essential Role of Ubiquitin and TSG101 Protein in Formation and Function of the Central Supramolecular Activation Cluster. Immunity (2010) 32:531–40. doi: 10.1016/j.immuni.2010.04.005 PMC2905630 20399684

[19] Alcover A Alarcón B Di Bartolo V. Cell Biology of T Cell Receptor Expression and Regulation. Annu Rev Immunol (2018) 36:103–25. doi: 10.1146/annurev-immunol-042617-053429 29261409

[20] Choudhuri K Llodrá J Roth EW Tsai J Gordo S Wucherpfennig KW. Polarized Release of T-Cell-Receptor-Enriched Microvesicles at the Immunological Synapse. Nature (2014) 507:118–23. doi: 10.1038/nature12951 PMC3949170 24487619

[21] Quintana A Kummerow C Junker C Becherer U Hoth M. Morphological Changes of T Cells Following Formation of the Immunological Synapse Modulate Intracellular Calcium Signals. Cell Calcium (2009) 45:109–22. doi: 10.1016/j.ceca.2008.07.003 18789821

[22] Baixauli F Martín-Cófreces NB Morlino G Carrasco YR Calabia-Linares C Veiga E. The Mitochondrial Fission Factor Dynamin-Related Protein 1 Modulates T-Cell Receptor Signalling at the Immune Synapse. EMBO J (2011) 30:1238–50. doi: 10.1038/emboj.2011.25 PMC3094108 21326213

[23] Cassioli C Onnis A Finetti F Capitani N Brunetti J Compeer EB. The Bardet-Biedl Syndrome Complex Component BBS1 Controls T Cell Polarity During Immune Synapse Assembly. J Cell Sci (2021) 134(16):jcs258462. doi: 10.1242/jcs.258462 34423835 PMC7613584

[24] Blumenthal D Burkhardt JK. Multiple Actin Networks Coordinate Mechanotransduction at the Immunological Synapse. J Cell Biol (2020) 219(2):e201911058. doi: 10.1083/jcb.201911058 31977034 PMC7041673

[25] Wang JC Hammer JA. The Role of Actin and Myosin in Antigen Extraction by B Lymphocytes. Semin Cell Dev Biol (2020) 102:90–104. doi: 10.1016/j.semcdb.2019.10.017 31862219 PMC8800146

[26] Kim H-R Jun C-D. T Cell Microvilli: Sensors or Senders? Front Immunol (2019) 10:1753. doi: 10.3389/fimmu.2019.01753 31417549 PMC6682677

[27] Jung Y Riven I Feigelson SW Kartvelishvily E Tohya K Miyasaka M. Three-Dimensional Localization of T-Cell Receptors in Relation to Microvilli Using a Combination of Superresolution Microscopies. Proc Natl Acad Sci USA (2016) 113:E5916–24. doi: 10.1073/pnas.1605399113 PMC5056101 27647916

[28] Cai E Marchuk K Beemiller P Beppler C Rubashkin MG Weaver VM. Visualizing Dynamic Microvillar Search and Stabilization During Ligand Detection by T Cells. Science (2017) 356(6338):eaal3118. doi: 10.1126/science.aal3118 28495700 PMC6364556

[29] Kim H-R Mun Y Lee K-S Park Y-J Park J-S Park J-H. T Cell Microvilli Constitute Immunological Synaptosomes That Carry Messages to Antigen-Presenting Cells. Nat Commun (2018) 9:3630. doi: 10.1038/s41467-018-06090-8 30194420 PMC6128830

[30] Sedwick CE Morgan MM Jusino L Cannon JL Miller J Burkhardt JK. TCR. LFA-1, and CD28 Play Unique and Complementary Roles in Signaling T Cell Cytoskeletal Reorganization. J Immunol Baltim Md 1950 (1999) 162:1367–75. 9973391

[31] Spitaler M Emslie E Wood CD Cantrell D. Diacylglycerol and Protein Kinase D Localization During T Lymphocyte Activation. Immunity (2006) 24:535–46. doi: 10.1016/j.immuni.2006.02.013 16713972

[32] Quann EJ Merino E Furuta T Huse M. Localized Diacylglycerol Drives the Polarization of the Microtubule-Organizing Center in T Cells. Nat Immunol (2009) 10:627–35. doi: 10.1038/ni.1734 19430478

[33] Le Floc’h A Huse M. Molecular Mechanisms and Functional Implications of Polarized Actin Remodeling at the T Cell Immunological Synapse. Cell Mol Life Sci CMLS (2015) 72:537–56. doi: 10.1007/s00018-014-1760-7 PMC4294954 25355055

[34] Comrie WA Burkhardt JK. Action and Traction: Cytoskeletal Control of Receptor Triggering at the Immunological Synapse. Front Immunol (2016) 7:68. doi: 10.3389/fimmu.2016.00068 27014258 PMC4779853

[35] Gomez TS Kumar K Medeiros RB Shimizu Y Leibson PJ Billadeau DD. Formins Regulate the Actin-Related Protein 2/3 Complex-Independent Polarization of the Centrosome to the Immunological Synapse. Immunity (2007) 26:177–90. doi: 10.1016/j.immuni.2007.01.008 PMC2836258 17306570

[36] Banerjee PP Pandey R Zheng R Suhoski MM Monaco-Shawver L Orange JS. Cdc42-Interacting Protein-4 Functionally Links Actin and Microtubule Networks at the Cytolytic NK Cell Immunological Synapse. J Exp Med (2007) 204:2305–20. doi: 10.1084/jem.20061893 PMC2118451 17785506

[37] Lasserre R Alcover A. Cytoskeletal Cross-Talk in the Control of T Cell Antigen Receptor Signaling. FEBS Lett (2010) 584:4845–50. doi: 10.1016/j.febslet.2010.09.001 20828561

[38] Piotrowski JT Gomez TS Schoon RA Mangalam AK Billadeau DD. WASH Knockout T Cells Demonstrate Defective Receptor Trafficking, Proliferation, and Effector Function. Mol Cell Biol (2013) 33:958–73. doi: 10.1128/MCB.01288-12 PMC3623087 23275443

[39] Chemin K Bohineust A Dogniaux S Tourret M Guégan S Miro F. Cytokine Secretion by CD4+ T Cells at the Immunological Synapse Requires Cdc42-Dependent Local Actin Remodeling But Not Microtubule Organizing Center Polarity. J Immunol Baltim Md 1950 (2012) 189:2159–68. doi: 10.4049/jimmunol.1200156 22821962

[40] Ritter AT Asano Y Stinchcombe JC Dieckmann NMG Chen B-C Gawden-Bone C. Actin Depletion Initiates Events Leading to Granule Secretion at the Immunological Synapse. Immunity (2015) 42:864–76. doi: 10.1016/j.immuni.2015.04.013 PMC4448150 25992860

[41] Ritter AT Kapnick SM Murugesan S Schwartzberg PL Griffiths GM Lippincott-Schwartz J. Cortical Actin Recovery at the Immunological Synapse Leads to Termination of Lytic Granule Secretion in Cytotoxic T Lymphocytes. Proc Natl Acad Sci USA (2017) 114:E6585–94. doi: 10.1073/pnas.1710751114 PMC5559056 28716933

[42] Rak GD Mace EM Banerjee PP Svitkina T Orange JS. Natural Killer Cell Lytic Granule Secretion Occurs Through a Pervasive Actin Network at the Immune Synapse. PloS Biol (2011) 9:e1001151. doi: 10.1371/journal.pbio.1001151 21931536 PMC3172191

[43] Brown ACN Oddos S Dobbie IM Alakoskela J-M Parton RM Eissmann P. Remodelling of Cortical Actin Where Lytic Granules Dock at Natural Killer Cell Immune Synapses Revealed by Super-Resolution Microscopy. PloS Biol (2011) 9:e1001152. doi: 10.1371/journal.pbio.1001152 21931537 PMC3172219

[44] Carisey AF Mace EM Saeed MB Davis DM Orange JS. Nanoscale Dynamism of Actin Enables Secretory Function in Cytolytic Cells. Curr Biol CB (2018) 28:489–502.e9. doi: 10.1016/j.cub.2017.12.044 29398219 PMC5835143

[45] Hammer JA. Immunology: Is Actin at the Lytic Synapse a Friend or a Foe? Curr Biol CB (2018) 28:R155–7. doi: 10.1016/j.cub.2018.01.013 PMC8796140 29462581

[46] Bornens M Moudjou M. Studying the Composition and Function of Centrosomes in Vertebrates. Methods Cell Biol (1999) 61:13–34. doi: 10.1016/s0091-679x(08)61973-1 9891307

[47] Andersen JS Wilkinson CJ Mayor T Mortensen P Nigg EA Mann M. Proteomic Characterization of the Human Centrosome by Protein Correlation Profiling. Nature (2003) 426:570–4. doi: 10.1038/nature02166 14654843

[48] Jakobsen L Vanselow K Skogs M Toyoda Y Lundberg E Poser I. Novel Asymmetrically Localizing Components of Human Centrosomes Identified by Complementary Proteomics Methods. EMBO J (2011) 30:1520–35. doi: 10.1038/emboj.2011.63 PMC3102290 21399614

[49] Firat-Karalar EN Sante J Elliott S Stearns T. Proteomic Analysis of Mammalian Sperm Cells Identifies New Components of the Centrosome. J Cell Sci (2014) 127:4128–33. doi: 10.1242/jcs.157008 PMC4179487 25074808

[50] Farina F Gaillard J Guérin C Couté Y Sillibourne J Blanchoin L. The Centrosome Is an Actin-Organizing Centre. Nat Cell Biol (2016) 18:65–75. doi: 10.1038/ncb3285 26655833 PMC4880044

[51] Obino D Farina F Malbec O Sáez PJ Maurin M Gaillard J. Actin Nucleation at the Centrosome Controls Lymphocyte Polarity. Nat Commun (2016) 7:10969. doi: 10.1038/ncomms10969 26987298 PMC4802043

[52] Luxton GWG Gomes ER Folker ES Vintinner E Gundersen GG. Linear Arrays of Nuclear Envelope Proteins Harness Retrograde Actin Flow for Nuclear Movement. Science (2010) 329:956–9. doi: 10.1126/science.1189072 PMC3938394 20724637

[53] Kim D-H Cho S Wirtz D. Tight Coupling Between Nucleus and Cell Migration Through the Perinuclear Actin Cap. J Cell Sci (2014) 127:2528–41. doi: 10.1242/jcs.144345 PMC4038945 24639463

[54] Kutscheidt S Zhu R Antoku S Luxton GWG Stagljar I Fackler OT. FHOD1 Interaction With Nesprin-2G Mediates TAN Line Formation and Nuclear Movement. Nat Cell Biol (2014) 16:708–15. doi: 10.1038/ncb2981 PMC4113092 24880667

[55] Kwon M Bagonis M Danuser G Pellman D. Direct Microtubule-Binding by Myosin-10 Orients Centrosomes Toward Retraction Fibers and Subcortical Actin Clouds. Dev Cell (2015) 34:323–37. doi: 10.1016/j.devcel.2015.06.013 PMC4672950 26235048

[56] Inoue D Obino D Pineau J Farina F Gaillard J Guerin C. Actin Filaments Regulate Microtubule Growth at the Centrosome. EMBO J (2019) 38(11):e99630. doi: 10.15252/embj.201899630 30902847 PMC6545561

[57] Bello-Gamboa A Velasco M Moreno S Herranz G Ilie R Huetos S. Actin Reorganization at the Centrosomal Area and the Immune Synapse Regulates Polarized Secretory Traffic of Multivesicular Bodies in T Lymphocytes. J Extracell Vesicles (2020) 9:1759926. doi: 10.1080/20013078.2020.1759926 32939232 PMC7480611

[58] Ibañez-Vega J Del Valle Batalla F Saez JJ Soza A Yuseff M-I. Proteasome Dependent Actin Remodeling Facilitates Antigen Extraction at the Immune Synapse of B Cells. Front Immunol (2019) 10:225. doi: 10.3389/fimmu.2019.00225 30873155 PMC6401660

[59] Ibañez-Vega J Del Valle F Sáez JJ Guzman F Diaz J Soza A. Ecm29-Dependent Proteasome Localization Regulates Cytoskeleton Remodeling at the Immune Synapse. Front Cell Dev Biol (2021) 9:650817. doi: 10.3389/fcell.2021.650817 34055780 PMC8155528

[60] Schaller MD. Paxillin: A Focal Adhesion-Associated Adaptor Protein. Oncogene (2001) 20:6459–72. doi: 10.1038/sj.onc.1204786 11607845

[61] López-Colomé AM Lee-Rivera I Benavides-Hidalgo R López E. Paxillin: A Crossroad in Pathological Cell Migration. J Hematol OncolJ Hematol Oncol (2017) 10:50. doi: 10.1186/s13045-017-0418-y 28214467 PMC5316197

[62] Herreros L Rodríguez-Fernandez JL Brown MC Alonso-Lebrero JL Cabañas C Sánchez-Madrid F. Paxillin Localizes to the Lymphocyte Microtubule Organizing Center and Associates With the Microtubule Cytoskeleton. J Biol Chem (2000) 275:26436–40. doi: 10.1074/jbc.M003970200 10840040

[63] Robertson LK Ostergaard HL. Paxillin Associates With the Microtubule Cytoskeleton and the Immunological Synapse of CTL Through Its Leucine-Aspartic Acid Domains and Contributes to Microtubule Organizing Center Reorientation. J Immunol Baltim Md 1950 (2011) 187:5824–33. doi: 10.4049/jimmunol.1003690 22043013

[64] Murugesan S Hong J Yi J Li D Beach JR Shao L. Formin-Generated Actomyosin Arcs Propel T Cell Receptor Microcluster Movement at the Immune Synapse. J Cell Biol (2016) 215:383–99. doi: 10.1083/jcb.201603080 PMC5100289 27799367

[65] Ramalingam N Zhao H Breitsprecher D Lappalainen P Faix J Schleicher M. Phospholipids Regulate Localization and Activity of Mdia1 Formin. Eur J Cell Biol (2010) 89:723–32. doi: 10.1016/j.ejcb.2010.06.001 20619927

[66] Baumeister W Walz J Zühl F Seemüller E. The Proteasome: Paradigm of a Self-Compartmentalizing Protease. Cell (1998) 92:367–80. doi: 10.1016/s0092-8674(00)80929-0 9476896

[67] Vora SM Phillips BT. The Benefits of Local Depletion: The Centrosome as a Scaffold for Ubiquitin-Proteasome-Mediated Degradation. Cell Cycle Georget Tex (2016) 15:2124–34. doi: 10.1080/15384101.2016.1196306 PMC4993542 27294844

[68] Yan D Guo L Wang Y. Requirement of Dendritic Akt Degradation by the Ubiquitin-Proteasome System for Neuronal Polarity. J Cell Biol (2006) 174:415–24. doi: 10.1083/jcb.200511028 PMC2064237 16864652

[69] Hsu M-T Guo C-L Liou AY Chang T-Y Ng M-C Florea BI. Stage-Dependent Axon Transport of Proteasomes Contributes to Axon Development. Dev Cell (2015) 35:418–31. doi: 10.1016/j.devcel.2015.10.018 26609957

[70] Spektor A Tsang WY Khoo D Dynlacht BD. Cep97 and CP110 Suppress a Cilia Assembly Program. Cell (2007) 130:678–90. doi: 10.1016/j.cell.2007.06.027 17719545

[71] Tuz K Bachmann-Gagescu R O’Day DR Hua K Isabella CR Phelps IG. Mutations in CSPP1 Cause Primary Cilia Abnormalities and Joubert Syndrome With or Without Jeune Asphyxiating Thoracic Dystrophy. Am J Hum Genet (2014) 94:62–72. doi: 10.1016/j.ajhg.2013.11.019 24360808 PMC3882733

[72] Kasahara K Kawakami Y Kiyono T Yonemura S Kawamura Y Era S. Ubiquitin-Proteasome System Controls Ciliogenesis at the Initial Step of Axoneme Extension. Nat Commun (2014) 5:5081. doi: 10.1038/ncomms6081 25270598 PMC4205846

[73] Liu YP Tsai I-C Morleo M Oh EC Leitch CC Massa F. Ciliopathy Proteins Regulate Paracrine Signaling by Modulating Proteasomal Degradation of Mediators. J Clin Invest (2014) 124:2059–70. doi: 10.1172/JCI71898 PMC4001542 24691443

[74] Gerhardt C Leu T Lier JM Rüther U. The Cilia-Regulated Proteasome and its Role in the Development of Ciliopathies and Cancer. Cilia (2016) 5:14. doi: 10.1186/s13630-016-0035-3 27293550 PMC4901515

[75] Chang JT Ciocca ML Kinjyo I Palanivel VR McClurkin CE Dejong CS. Asymmetric Proteasome Segregation as a Mechanism for Unequal Partitioning of the Transcription Factor T-Bet During T Lymphocyte Division. Immunity (2011) 34:492–504. doi: 10.1016/j.immuni.2011.03.017 21497118 PMC3088519

[76] Widjaja CE Olvera JG Metz PJ Phan AT Savas JN de Bruin G. Proteasome Activity Regulates CD8+ T Lymphocyte Metabolism and Fate Specification. J Clin Invest (2017) 127:3609–23. doi: 10.1172/JCI90895 PMC5617668 28846070

[77] Martin-Cofreces NB Chichon FJ Calvo E Torralba D Bustos-Moran E Dosil SG. The Chaperonin CCT Controls T Cell Receptor-Driven 3D Configuration of Centrioles. Sci Adv (2020) 6(49):eabb7242. doi: 10.1126/sciadv.abb7242 33268369 PMC7821906

[78] Fokin AI Gautreau AM. Assembly and Activity of the WASH Molecular Machine: Distinctive Features at the Crossroads of the Actin and Microtubule Cytoskeletons. Front Cell Dev Biol (2021) 9:658865. doi: 10.3389/fcell.2021.658865 33869225 PMC8047104

[79] Hao Y-H Doyle JM Ramanathan S Gomez TS Jia D Xu M. Regulation of WASH-Dependent Actin Polymerization and Protein Trafficking by Ubiquitination. Cell (2013) 152:1051–64. doi: 10.1016/j.cell.2013.01.051 PMC3640276 23452853

[80] Didier C Merdes A Gairin J-E Jabrane-Ferrat N. Inhibition of Proteasome Activity Impairs Centrosome-Dependent Microtubule Nucleation and Organization. Mol Biol Cell (2008) 19:1220–9. doi: 10.1091/mbc.e06-12-1140 PMC2262974 18094058

[81] Nachury MV. The Molecular Machines That Traffic Signaling Receptors Into and Out of Cilia. Curr Opin Cell Biol (2018) 51:124–31. doi: 10.1016/j.ceb.2018.03.004 PMC5949257 29579578

[82] Nakayama K Katoh Y. Ciliary Protein Trafficking Mediated by IFT and BBSome Complexes With the Aid of Kinesin-2 and Dynein-2 Motors. J Biochem (Tokyo) (2018) 163:155–64. doi: 10.1093/jb/mvx087 29272450

[83] Cassioli C. Baldari CT. A Ciliary View of the Immunological Synapse. Cells (2019) 8(8):789. doi: 10.3390/cells8080789 PMC6721628 31362462

[84] Douanne T Stinchcombe JC Griffiths GM. Teasing Out Function From Morphology: Similarities Between Primary Cilia and Immune Synapses. J Cell Biol (2021) 220(6):e202102089. doi: 10.1083/jcb.202102089 33956049 PMC8105739

[85] Hernandez-Hernandez V Pravincumar P Diaz-Font A May-Simera H Jenkins D Knight M. Bardet-Biedl Syndrome Proteins Control the Cilia Length Through Regulation of Actin Polymerization. Hum Mol Genet (2013) 22:3858–68. doi: 10.1093/hmg/ddt241 PMC3766180 23716571

[86] Maskalenko N Nath S Ramakrishnan A Anikeeva N Sykulev Y Poenie M. The DISC1-Girdin Complex - A Missing Link in Signaling to the T Cell Cytoskeleton. J Cell Sci (2020) 133(13):jcs242875. doi: 10.1242/jcs.242875 32482796 PMC7358132

[87] Yi J Wu X Chung AH Chen JK Kapoor TM Hammer JA. Centrosome Repositioning in T Cells Is Biphasic and Driven by Microtubule End-on Capture-Shrinkage. J Cell Biol (2013) 202:779–92. doi: 10.1083/jcb.201301004 PMC3760611 23979719

[88] Combs J Kim SJ Tan S Ligon LA Holzbaur ELF Kuhn J. Recruitment of Dynein to the Jurkat Immunological Synapse. Proc Natl Acad Sci USA (2006) 103:14883–8. doi: 10.1073/pnas.0600914103 PMC1595445 16990435

[89] Martín-Cófreces NB Robles-Valero J Cabrero JR Mittelbrunn M Gordón-Alonso M Sung C-H. MTOC Translocation Modulates IS Formation and Controls Sustained T Cell Signaling. J Cell Biol (2008) 182:951–62. doi: 10.1083/jcb.200801014 PMC2528574 18779373

[90] Hooikaas PJ Damstra HG Gros OJ van Riel WE Martin M Smits YT. Kinesin-4 KIF21B Limits Microtubule Growth to Allow Rapid Centrosome Polarization in T Cells. eLife (2020) 9:e62876. doi: 10.7554/eLife.62876 33346730 PMC7817182

[91] Nejmeddine M Negi VS Mukherjee S Tanaka Y Orth K Taylor GP. HTLV-1-Tax and ICAM-1 Act on T-Cell Signal Pathways to Polarize the Microtubule-Organizing Center at the Virological Synapse. Blood (2009) 114:1016–25. doi: 10.1182/blood-2008-03-136770 19494354

[92] Tsun A Qureshi I Stinchcombe JC Jenkins MR de la Roche M Kleczkowska J. Centrosome Docking at the Immunological Synapse is Controlled by Lck Signaling. J Cell Biol (2011) 192:663–74. doi: 10.1083/jcb.201008140 PMC3044125 21339332

